# Vision-Based Environmental Sensing for Flood Risk Forecasting: Dataset Relabeling and Temporal Multi-Task Learning

**DOI:** 10.3390/s26113520

**Published:** 2026-06-02

**Authors:** Seungju Lee, Gooman Park

**Affiliations:** Department of Smart ICT Convergence Engineering, Seoul National University of Science and Technology, Seoul 01811, Republic of Korea; sjleee05@gmail.com

**Keywords:** flood prediction, CCTV, multimodal learning, semantic segmentation, time-series forecasting, risk estimation

## Abstract

River flooding and urban inundation require forecasting systems that can anticipate future risk, rather than systems that only estimate the current water state. However, real-world closed-circuit television (CCTV)-based flood datasets often contain imbalanced or temporally inconsistent risk labels. In addition, most image-based approaches remain limited to static scene understanding. This study proposes a dataset reformulation and temporal multi-task forecasting framework for CCTV-based flood-risk prediction. First, we introduce a site-relative relabeling strategy that converts noisy frame-level danger annotations into four risk levels using visual flood indicators and lightweight environmental cues. Second, we transform the original frame-based dataset into site-hour sequences for multi-horizon forecasting at 1 h, 3 h, and 6 h. Third, we evaluate image-only, weather-only, and naive multimodal configurations to examine the role and limitations of heterogeneous sensor fusion. On the reformulated dataset, the image-only temporal model achieved the best overall performance, with a mean Intersection over Union (mIoU) of 0.892, Dice score of 0.940, macro-averaged F1 score (Macro-F1) of 0.532, and high-risk recall of 0.642. In contrast, naive multimodal fusion reduced Macro-F1 to 0.267 and high-risk recall to 0.070. This result indicates that additional weather inputs do not automatically improve prediction when cross-modal signals are noisy, weakly correlated, or temporally misaligned. The ablation results further showed that removing temporal modeling decreased Macro-F1 to 0.227 and high-risk recall to 0.000. These findings demonstrate that dataset reformulation and temporal modeling are essential for extending CCTV-based flood analysis from static estimation to future risk forecasting. They also suggest that robust cross-modal alignment is required before multimodal sensing can provide reliable performance gains.

## 1. Introduction

River flooding and urban inundation are among the most critical natural hazards, and their frequency and intensity have increased under climate change and extreme weather conditions [1]. These events cause substantial human casualties and economic losses, highlighting the need for proactive flood-risk prediction frameworks rather than purely reactive monitoring systems [1,2].

Conventional river water level prediction systems primarily rely on sensor-based measurements at specific locations. While these approaches can provide high accuracy, they suffer from high installation and maintenance costs, as well as limited spatial coverage. With recent advances in deep learning, computer vision, and sensor-based monitoring, image-based flood analysis has emerged as a promising alternative for flood monitoring and water level estimation [3,4,5,6]. CCTV imagery offers wide-area coverage and enables data acquisition without additional sensor deployment.

However, existing image-based flood-monitoring approaches are typically limited to current-state estimation, such as detecting flooded regions, segmenting water areas, or estimating water levels from visual observations [3,4,5,6]. In practical disaster-response scenarios, anticipating flood risk over the next few hours is often more important than assessing only the current condition. This requires temporal modeling of visual observations, together with environmental factors such as rainfall, temperature, and atmospheric pressure, which are widely used in hydrological forecasting [1,2].

Furthermore, real-world flood-risk datasets can contain noisy, imbalanced, or temporally inconsistent labels, which may lead to biased learning and poor recognition of minority or high-risk cases [7]. In the dataset used in this study, the original danger_level annotations are highly imbalanced and not directly organized for temporal forecasting, motivating a dataset reformulation step before model training.

To address these challenges, we first reconsider the structure of the CCTV-based flood dataset, rather than assuming that the original frame-level labels are directly suitable for forecasting. Specifically, we introduce a site-relative relabeling strategy to reconstruct risk levels using visual and environmental cues, and we reformulate static frame-level records into a temporal forecasting task. Within this reformulated setting, we evaluate image-only, weather-only, and multimodal configurations to determine whether heterogeneous sensing modalities provide complementary information. Importantly, the results show that naive multimodal fusion does not automatically improve flood-risk prediction and may degrade high-risk detection when weather observations are noisy, weakly correlated, or temporally misaligned with visual evidence.

In this context, CCTV cameras can be interpreted as visual sensors that continuously monitor environmental conditions, while meteorological measurements from Automatic Weather Stations (AWS) provide complementary environmental sensing signals. Therefore, flood-risk prediction can be viewed as a sensor fusion problem that integrates heterogeneous sensing modalities. However, effectively leveraging such heterogeneous sensor data remains challenging because naive fusion may fail when modalities differ in temporal resolution, noise level, or predictive strength [8,9].

The main contributions of this paper are summarized as follows. First, we propose a site-relative relabeling strategy that transforms noisy and imbalanced frame-level danger annotations into temporally meaningful risk levels. Second, we reformulate static CCTV records into site-hour sequences, enabling multi-horizon flood-risk forecasting rather than current-state estimation. Third, we establish a temporal multi-task framework for joint semantic segmentation and danger-level forecasting, and evaluate image-only, weather-only, and naive multimodal configurations. Finally, we provide quantitative and qualitative analyses showing that temporal modeling is essential, while naive multimodal fusion can underperform under noisy or misaligned environmental observations.

The remainder of this paper is organized as follows. Section 2 reviews related studies on vision-based flood monitoring, time-series flood prediction, and multimodal learning. Section 3 describes the dataset, relabeling strategy, sequence reformulation, and temporal multi-task forecasting framework. Section 4 presents the experimental setup, quantitative results, ablation study, sensitivity analysis, and qualitative case study. Section 5 and Section 6 discuss the findings and conclude the paper, respectively.

## 2. Related Work

### 2.1. Vision-Based Water Level Estimation

Vision-based water level estimation has primarily relied on semantic segmentation techniques to detect water regions in images. These approaches enable precise modeling of the spatial distribution of water and have been applied to flood monitoring, visual water level estimation, and surveillance-camera-based environmental sensing [3,5,6]. However, they are inherently limited to static state estimation and lack the capability to capture temporal dynamics or predict future conditions.

Representative segmentation-based methods include Mask R-CNN, U-Net, Fully Convolutional Networks (FCNs), and SegFormer [10,11,12,13]. In addition, DeepLab variants and pyramid pooling-based architectures have been widely adopted to improve segmentation performance [14,15].

More recently, the performance of semantic segmentation has significantly improved with the introduction of advanced architectures such as Vision Transformer (ViT) [16], Swin Transformer [17], HRNet [18], and UNet++ [19]. Despite these advances, most existing methods focus on pixel-level understanding at a single time step and do not address future risk prediction.

### 2.2. Time-Series-Based Flood Prediction

In the field of flood and water level prediction, various approaches based on time-series modeling have been proposed. Recurrent neural networks such as Long Short-Term Memory (LSTM), Transformer-based models, and convolutional time-series models have been widely used [20,21,22]. These methods typically rely on sequential inputs such as rainfall measurements and water level sensor data.

In addition, hydrological approaches such as rainfall-runoff modeling have also incorporated time-series learning techniques [1,2]. More advanced models, including ConvLSTM [23] and other deep temporal prediction architectures [24,25], have been applied to environmental and hydrological forecasting tasks. While these methods effectively capture temporal dependencies, they often lack visual context, limiting their ability to model complex real-world flooding scenarios.

### 2.3. Multimodal and Multi-Task Learning

Multimodal learning has recently gained significant attention as a means to integrate heterogeneous data sources. Models such as VisualBERT, ViLBERT, and CLIP demonstrate the effectiveness of combining multiple modalities, including vision and language, to improve performance [26,27,28]. In addition, various multimodal fusion strategies have been proposed to better model cross-modal interactions.

Multi-task learning has also been shown to improve performance and generalization by jointly learning multiple related tasks within a single model [29]. Recent studies further highlight the importance of effectively modeling interactions between modalities in multimodal settings [8,9]. In particular, multimodal learning can be interpreted as a sensor fusion problem, which has been widely studied in sensing systems where heterogeneous sensor data must be effectively aligned and fused.

### 2.4. Limitations of Existing Approaches

Despite these advances, most existing studies are limited to single-modality inputs or focus primarily on current-state estimation. In real-world CCTV-based environments, future flood-risk prediction requires not only temporal modeling but also reliable alignment between visual observations and environmental sensor measurements. Prior studies on multimodal learning have shown that simple feature concatenation may fail when modalities have different noise levels, temporal resolutions, or predictive strengths [8,9]. In addition, learning from noisy or imbalanced labels can lead to biased decision boundaries and poor minority-class recognition, particularly in high-risk prediction tasks [7]. However, limited work has jointly addressed dataset quality, temporal forecasting, and multimodal sensor fusion in CCTV-based flood-risk prediction. This gap motivates our dataset reformulation and temporal multi-task analysis.

## 3. Materials and Methods

### 3.1. Dataset Description

We used the Busan Flood Risk Composite Dataset provided by AIHUB [30]. The dataset includes closed-circuit television (CCTV) images, Common Objects in Context (COCO)-format semantic segmentation annotations, flood-risk metadata, and meteorological observations obtained from Automatic Weather Stations (AWS) operated by the Korea Meteorological Administration [31]. In this study, CCTV images are treated as visual sensor observations, whereas AWS measurements are treated as environmental sensor observations.

**Figure 1:**
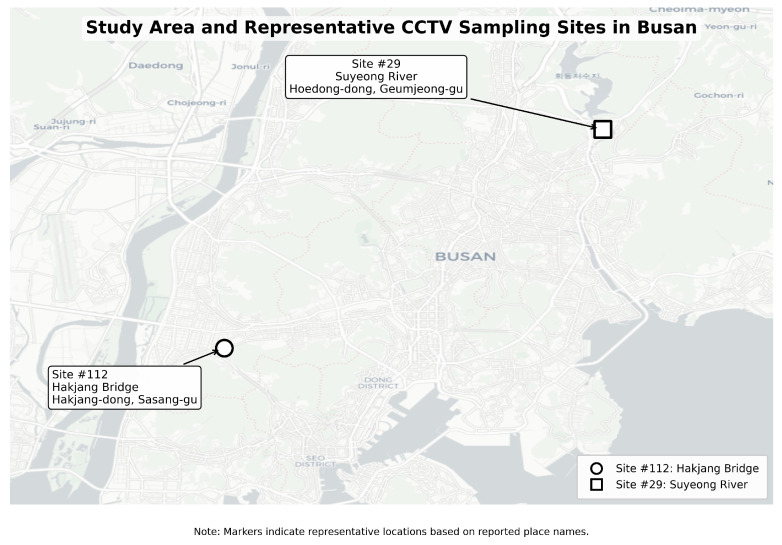
Study area and representative CCTV sampling sites used in the Busan Flood Risk Composite Dataset. Site #112 indicates Hakjang Bridge in Hakjang-dong, Sasang-gu, Busan, and Site #29 indicates the Suyeong River area in Hoedong-dong, Geumjeong-gu, Busan. These two sites are highlighted as representative locations. Korean place names appearing in the figure denote local administrative or river-location names and do not affect the scientific interpretation of the figure.

Figure 1 shows the geographical distribution of the representative CCTV sampling sites in the study area. The sampling sites are located in Busan, Republic of Korea, and represent real-world urban flood-monitoring environments. In particular, Site #112 corresponds to Hakjang Bridge in Hakjang-dong, Sasang-gu, while Site #29 corresponds to the Suyeong River area in Hoedong-dong, Geumjeong-gu. These two sites are explicitly highlighted because they are used as representative locations in the qualitative and failure-case analyses.

The original dataset contains 2861 frame-level samples collected from multiple CCTV sites [30]. Each sample includes an image, segmentation annotations for flood-related semantic regions, and metadata such as danger_level, river_level, and observational_water_level. However, the original frame-level structure is not directly suitable for future risk forecasting because the danger labels are highly imbalanced, key water level observations are largely missing, and frame-level labels are not temporally organized.

### 3.2. Limitations of the Original Labels

The original dataset exhibits three critical limitations for future flood-risk forecasting, as summarized in Table 1. First, the danger_level labels are highly concentrated on a small number of classes, making meaningful multi-class risk learning difficult. Second, the observational_water_level values are largely missing, which prevents direct supervised learning based on measured water levels. Third, the original frame-level annotations are not temporally organized and may remain constant or fluctuate inconsistently within the same site-hour interval. These characteristics encourage the model to learn trivial or unstable decision patterns rather than temporally meaningful risk transitions.

As summarized in Table 2, the original dataset follows a static frame-based structure that does not adequately support temporal forecasting. Such characteristics encourage the model to converge to trivial solutions rather than learning meaningful risk representations. Therefore, dataset reformulation is required before training a future risk prediction model.

### 3.3. Relabeling Strategy

**Figure 2:**
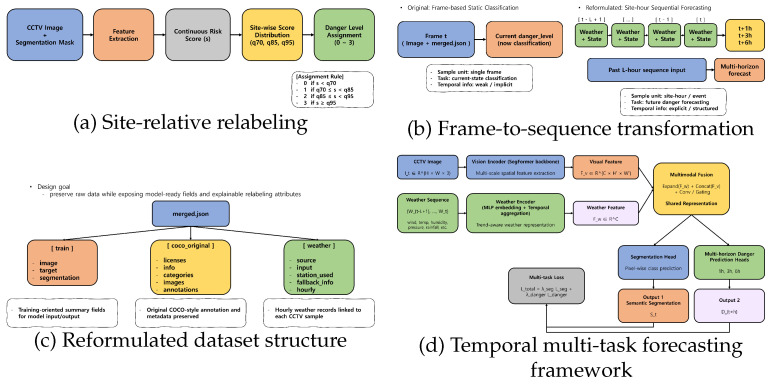
Overall pipeline of the proposed CCTV-based flood-risk forecasting framework. The sub-figures are enlarged to improve the readability of the workflow components. (**a**) Site-relative relabeling converts noisy frame-level annotations into risk-aware danger levels. (**b**) Frame-level records are transformed into site-hour sequences for multi-horizon forecasting. (**c**) The reformulated dataset integrates train-ready samples, original COCO annotations, and time-aligned weather observations. (**d**) The temporal multi-task framework jointly performs current-frame semantic segmentation and future danger-level prediction.

To address these issues, we propose a relabeling strategy based on *site-relative risk modeling*. The overall workflow of the proposed dataset reformulation and temporal multi-task forecasting framework is summarized in Figure 2. The relabeling process is specifically illustrated in Figure 2a.

We first extract visual and environmental features from CCTV images, segmentation results, and available metadata. These features include the water area ratio, waterline height, rainfall intensity, river-level context, and spatial overlap between key semantic regions. Together, they capture both scene-level visual evidence and environmental conditions related to flood risk.

Inspired by prior segmentation-based water-region analysis and hydrological forecasting studies [1,2,3,6], we define an empirical risk score that combines visible water extent, waterline position, rainfall intensity, river-level context, and semantic overlap with vulnerable regions:

(1) $$s=3.0r_{w}+2.0h_{w}+0.3r_{\mathrm{rain}}+0.2r_{\mathrm{river}}+r_{\mathrm{overlap}}.$$

where each component is defined as follows:

- Water Area Ratio: $r_{w}=\frac{A_{\mathrm{water}}}{A_{\mathrm{image}}}$.
- Waterline Height: $h_{w}=1-\frac{y_{\min}^{\mathrm{water}}}{H}$.
- Rainfall Boost:
  The rainfall term follows the common assumption in hydrological forecasting that recent and accumulated precipitation provide useful short-term risk indicators [1,2]:
  (2) $$r_{\mathrm{rain}}=\log\left(1+R_{1h}\right)+0.5\log\left(1+R_{3h}\right)+0.2\log\left(1+R_{10m}\right).$$
- River Level Boost:
  The river-level term provides additional environmental context when river observations are available, following hydrological forecasting studies that use water level measurements as key predictors of flood risk [1,2]:
  (3) $$r_{\mathrm{river}}=0.5\log\left(1+L_{\mathrm{river}}\right).$$
- Overlap Boost:
  Finally, the overlap term captures whether the detected water region intrudes into semantically vulnerable areas using Intersection over Union (IoU), following the intuition of segmentation-based flood-state interpretation [3,6]:
  (4) $$r_{\mathrm{overlap}}=10\cdot \mathrm{IoU}\left(\mathrm{water},\mathrm{driveway}\right)+3\cdot \mathrm{IoU}\left(\mathrm{water},\mathrm{waterside}\right).$$

This relabeling strategy is not intended to replace physically calibrated hydrological measurements. Rather, it serves as an empirical and interpretable proxy for converting noisy CCTV annotations into learnable risk categories. Because the original observational water level values are largely missing and the danger-level labels are severely imbalanced, directly training a forecasting model on the original labels may lead to trivial or unstable learning. Therefore, the proposed score combines visually grounded indicators with lightweight environmental context to produce temporally meaningful targets for short-term risk forecasting.

The weights in Equation (1) were assigned based on empirical observations and domain knowledge regarding the reliability and directness of each cue. To reduce the arbitrariness of this empirical design, we further evaluate alternative visual and weather weighting schemes in Section 4.7 and report their effect on Macro-F1 and high-risk recall. The water area ratio receives the largest weight because the visible extent of water is the most direct image-based indicator of inundation in CCTV scenes. The waterline height is also strongly weighted because upward movement of the water boundary provides an interpretable proxy for increasing flood risk. Rainfall and river-level terms are assigned smaller weights because they provide contextual environmental information, but their relationship with the CCTV-visible flood state can be indirect, delayed, or affected by site-specific drainage and river geometry. The overlap term is included as a high-risk visual cue because water intrusion into driveway or waterside regions indicates spatial expansion of water into semantically vulnerable areas.

To convert this continuous score into discrete risk levels, we apply a quantile-based strategy within each CCTV site:

- d=0 if $s<q_{70}$.
- d=1 if $q_{70}\leq s<q_{85}$.
- d=2 if $q_{85}\leq s<q_{95}$.
- d=3 if $s\geq q_{95}$.

This site-relative approach effectively normalizes environmental variations across different locations and produces more stable and meaningful labels compared to global threshold-based methods. The effectiveness of relabeling is demonstrated in Table 3.

### 3.4. Dataset Transformation

The original dataset is organized as a frame-level classification problem. We reformulate it into a sequence-based forecasting task, as illustrated in Figure 2b. Instead of predicting risk from a single frame, the model uses a sequence of past observations over *L* time steps as input and predicts future risk levels at multiple horizons, including 1 h, 3 h, and 6 h. This transformation enables the model to capture temporal patterns and risk transitions that cannot be represented by isolated frames.

### 3.5. Final Dataset Structure

The final dataset is organized into a merged structure consisting of three components: train, coco_original, and weather, as shown in Figure 2c. The train component contains model-ready inputs and targets, while coco_original preserves the original annotations and metadata. The weather component provides time-aligned meteorological data. This design maintains data integrity while enabling efficient multimodal learning.

### 3.6. Dataset Statistics

The original dataset consists of 2861 frames. After reformulation, it is reorganized into 400 site-hour events and 230 sequence samples. Although the resulting number of sequences is relatively small, the reformulation changes the learning problem from redundant frame-level classification to temporally structured forecasting. Therefore, the reduced sample count should be interpreted as a trade-off for temporal consistency and leakage prevention rather than a simple loss of data volume. As shown in Table 3, the class distribution becomes more balanced compared to the original dataset, enabling more meaningful training and evaluation of future danger-level prediction.

The reformulated dataset provides four practical advantages over the original frame-level structure: reduced label noise, improved temporal consistency, support for future risk prediction, and enhanced interpretability through explicit feature design. These properties make the dataset more suitable for evaluating short-term flood-risk forecasting models.

### 3.7. Problem Definition

Let $I_{t}$ denote the CCTV image observed at time *t*, and let $W_{t-L+1:t}=\left\{W_{t-L+1},\ldots ,W_{t}\right\}$ denote a sequence of past weather observations over a temporal window of length *L*. The objective is to jointly estimate the current semantic segmentation map $S_{t}$ and future danger levels $D_{t+h}$ at multiple prediction horizons h∈{1,3,6}:

(5) $$\left(S_{t},{\left\{D_{t+h}\right\}}_{h\in \{1,3,6\}}\right)=f_{\theta }\left(I_{t},W_{t-L+1:t}\right).$$

Depending on the input configuration, the model operates in one of three settings:

(6) $$f_{\theta }=\left\{\begin{array}{cc} f_{\theta }^{\mathrm{img}}\left(I_{t}\right), & \mathrm{image}\text{-}\mathrm{only},\\ f_{\theta }^{w}\left(W_{t-L+1:t}\right), & \mathrm{weather}\text{-}\mathrm{only},\\ f_{\theta }^{\mathrm{mm}}\left(I_{t},W_{t-L+1:t}\right), & \mathrm{multimodal}. \end{array}\right.$$

### 3.8. Temporal Multi-Task Forecasting Framework

As illustrated in Figure 2d, the proposed framework jointly performs current-frame semantic segmentation and future danger-level prediction. The image stream adopts a SegFormer backbone [13] to extract spatial features from CCTV images, whereas the weather stream uses a multilayer perceptron (MLP)-based temporal aggregation module to encode meteorological observations. In the multimodal setting, visual and weather features are concatenated and projected into a shared representation. This representation is then passed to the segmentation head and the horizon-specific danger prediction heads.

Table 4 summarizes the implementation configuration of the proposed framework, including the encoder architectures, fusion strategy, task heads, and forecasting horizons.

## 4. Experiments and Results

### 4.1. Experimental Setup

Experiments were conducted on the reformulated site-hour sequence dataset. To prevent spatial leakage between training and validation data, the split was performed at the CCTV-site level. This site-level split was used to evaluate cross-site generalization rather than frame-level memorization. All models were trained using an input image resolution of 1024×1024 and optimized with AdamW [32].

We evaluated three input configurations: image-only, weather-only, and naive multimodal fusion. The image-only setting was used to assess the predictive value of visual flood cues, the weather-only setting was used to examine the standalone contribution of meteorological observations, and the multimodal setting was used to test whether simple feature-level fusion provides complementary gains.

### 4.2. Evaluation Metrics

Segmentation performance was evaluated using mean Intersection over Union (mIoU) and Dice score, which is widely used to measure region overlap in dense prediction tasks [33].

For danger-level prediction, we used the Macro-F1 score, accuracy, and Mean Absolute Error (MAE). Additionally, to evaluate performance in critical scenarios, we defined *high-risk* as classes greater than or equal to 2 and computed high-risk recall.

### 4.3. Quantitative Results

Table 5 presents the overall performance comparison among image-only, weather-only, and multimodal configurations.

For semantic segmentation, the image-only model achieved the best performance, with an mIoU of 0.892 and a Dice score of 0.940. The multimodal model achieved comparable segmentation performance, with an mIoU of 0.884 and a Dice score of 0.936. These results indicate that visual information provides the dominant signal for capturing spatial flood patterns.

For danger-level prediction, the image-only temporal model achieved the best performance, with a Macro-F1 of 0.532, accuracy of 0.637, MAE of 0.498, and high-risk recall of 0.642. Compared with the image-only model, the naive multimodal model reduced Macro-F1 from 0.532 to 0.267, corresponding to a relative decrease of approximately 49.8%. More critically, high-risk recall decreased from 0.642 to 0.070, indicating that simple feature-level fusion substantially degraded the detection of high-risk cases. The weather-only model also showed limited predictive performance, with a Macro-F1 of 0.289 and high-risk recall of 0.205. These results suggest that CCTV-derived visual cues provide more direct and reliable evidence for short-term flood-risk prediction than the available AWS observations in the current dataset. Therefore, the multimodal results should not be interpreted as evidence against multimodal sensing itself. Instead, they indicate that naive feature-level fusion is insufficient when environmental observations are noisy, weakly correlated, or temporally misaligned with visual flood responses.

### 4.4. Temporal Consistency Analysis

To further verify the effect of dataset reformulation, we analyzed temporal consistency before and after relabeling. In the original frame-level annotations, danger levels frequently remained unchanged despite visible changes in water regions or fluctuated within the same site-hour interval. Such behavior indicates that the original labels were not sufficiently aligned with the temporal evolution of visible flood conditions.

After site-hour aggregation and quantile-based relabeling, the labels showed smoother temporal transitions and better correspondence with visible water expansion. This improvement is important because future risk prediction requires labels that evolve consistently over time rather than isolated frame-level annotations. As summarized in Table 6, the reformulated dataset provides a more suitable target structure for short-term forecasting despite the reduced number of training samples.

### 4.5. Horizon-Wise Analysis

**Figure 3:**
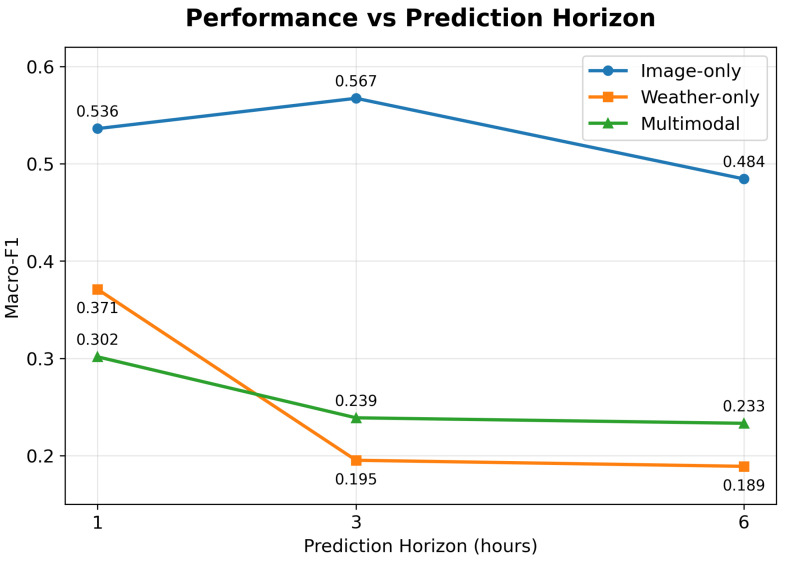
Macro-F1 score across prediction horizons for image-only, weather-only, and multimodal configurations. The image-only temporal model consistently outperforms the other settings across all horizons, while naive multimodal fusion does not provide complementary gains. This result directly supports our main finding that visual temporal cues are more reliable than noisy or weakly aligned weather features in the current dataset, and that simple feature-level fusion is insufficient for robust flood risk forecasting.

Figure 3 shows that the image-only temporal model maintains the highest Macro-F1 across all prediction horizons. This trend indicates that the performance gap is not limited to a single forecasting time point, but persists across the entire multi-horizon task. The lower performance of the multimodal model suggests that weather features, when fused through simple concatenation, do not provide stable complementary information and may instead interfere with the visual representation.

As the prediction horizon increases, performance gradually decreases for all models, reflecting the increased difficulty of long-term forecasting. The image-only model consistently outperforms other models, achieving Macro-F1 scores of 0.567, 0.567, and 0.484 for 1 h, 3 h, and 6 h, respectively.

**Figure 4:**
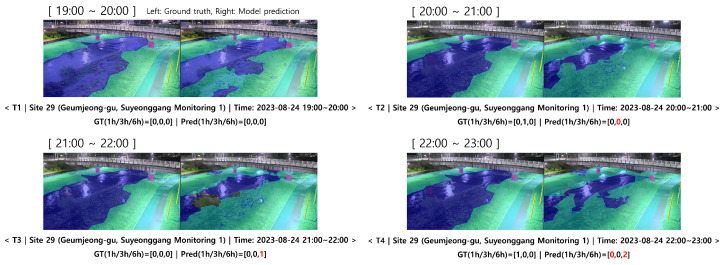
Temporal prediction examples across multiple horizons. The visualization shows that short-term predictions remain relatively stable when visual water patterns are consistent, whereas longer-horizon predictions become more uncertain. This supports the need for temporal modeling and illustrates the increasing difficulty of forecasting future danger levels beyond immediate visual evidence. Incorrect horizon-wise predictions are highlighted in red.

Figure 4 further illustrates how prediction reliability changes across horizons. The short-term predictions are relatively consistent with the visible water extent, whereas longer-horizon predictions become less stable. This qualitative trend supports the quantitative results in Figure 3 and reinforces the claim that temporal structure is essential for extending CCTV-based analysis from current-state estimation to future risk forecasting [25].

### 4.6. Ablation Study

We conducted ablation experiments to analyze the impact of temporal modeling and modality configuration. The results are summarized in Table 7.

Removing temporal modeling resulted in the largest degradation, reducing Macro-F1 from 0.532 to 0.227 and high-risk recall from 0.642 to 0.000. This indicates that the non-temporal baseline failed to detect high-risk cases. Therefore, the improvement cannot be attributed solely to the relabeling procedure or model capacity; it also depends on the temporal organization of the dataset and the ability to learn time-dependent risk transitions. In addition, the large gap between the image-only temporal model and the multimodal model suggests that the current fusion strategy may overfit to noisy environmental features or dilute the strong visual representation learned from CCTV images. The weather-only model showed limited performance, whereas the image-only temporal model achieved the best results, with a Macro-F1 of 0.532 and high-risk recall of 0.642.

### 4.7. Sensitivity Analysis of Relabeling Weights

To examine whether the proposed relabeling strategy is overly dependent on a specific set of heuristic weights, we additionally performed a sensitivity analysis by varying the relative contribution of visual and environmental terms. The goal of this analysis was not to optimize the weights exhaustively, but to verify whether the visual-dominant structure of the risk score provides stable and interpretable labels. The sensitivity analysis was conducted under the same evaluation protocol but focuses on the effect of relabeling-weight variants rather than on comparing model configurations. As shown in Table 8, the default visual-dominant weighting achieved the best balance between Macro-F1 and high-risk recall.

Reducing or increasing the weather contribution degraded performance, indicating that environmental cues are useful as auxiliary context but should not dominate the relabeling process. The visual-only score also showed lower performance, suggesting that lightweight weather information still contributes to constructing more informative risk labels. These results support the empirical design of Equation (1), where visual cues are treated as primary indicators and meteorological variables are incorporated as secondary contextual factors.

### 4.8. Qualitative Case Study

We conducted a qualitative case study to examine whether the quantitative trends are reflected in individual CCTV sequences. The case study focuses on two aspects: (1) whether the segmentation output captures visually meaningful flood-related regions, and (2) whether image-only and multimodal configurations produce different horizon-wise danger predictions under the same visual input.

**Figure 5:**
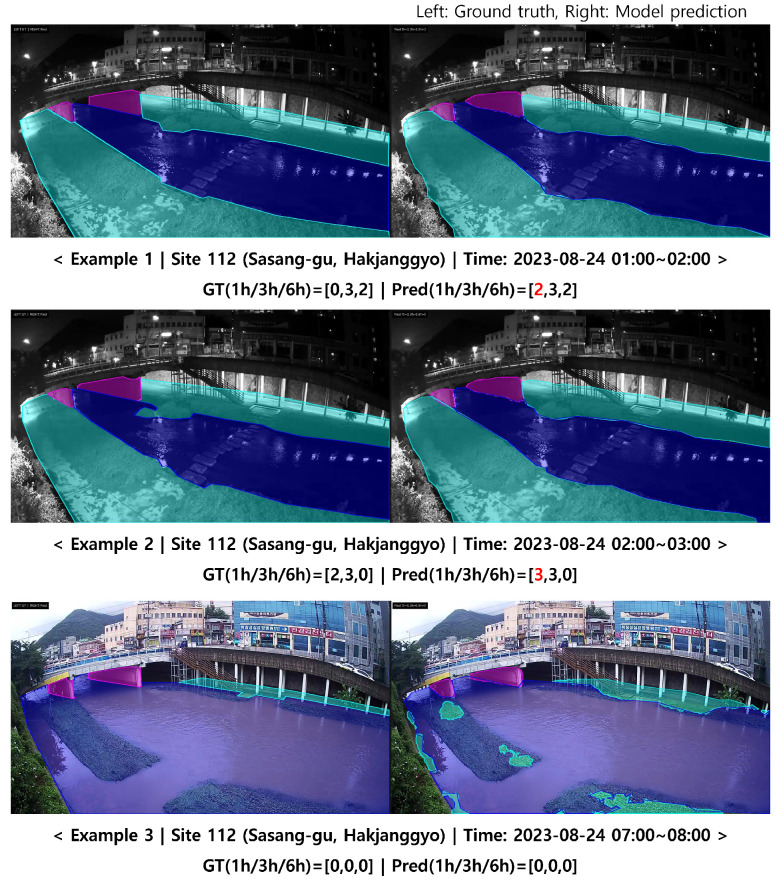
Qualitative segmentation results on the test dataset. Each example includes the CCTV site ID, timestamp, and multi-horizon ground-truth and predicted danger levels at 1 h, 3 h, and 6 h. Red text indicates horizons where the predicted danger level differs from the ground truth. The results show that the image-only temporal model accurately captures water regions and boundary structures, and that these visual cues provide meaningful evidence for future danger-level prediction.

Figure 5 shows representative segmentation and forecasting examples. Each case includes the CCTV site ID, timestamp, ground-truth danger levels, and predicted danger levels at 1 h, 3 h, and 6 h. Red annotations indicate horizons where the predicted danger level differs from the ground truth. The results show that the model generally captures water regions and boundary structures, and that these visual cues provide useful evidence for short-term danger-level prediction.

**Figure 6:**
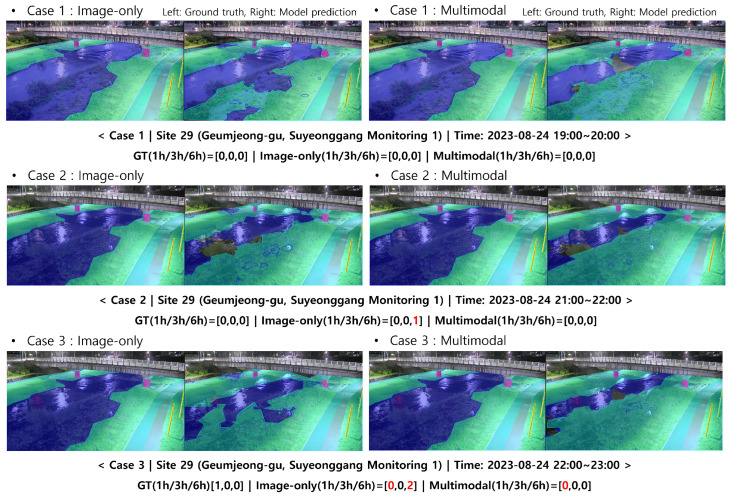
Qualitative comparison of image-only and multimodal predictions. Each case includes the CCTV site ID, timestamp, and multi-horizon ground-truth, image-only, and multimodal danger predictions at 1 h, 3 h, and 6 h. Red text indicates horizons where the predicted danger level differs from the ground truth. The examples illustrate that the two configurations can produce different prediction errors under the same visual input, highlighting the sensitivity of naive fusion to additional weather features.

Figure 6 compares image-only and multimodal predictions under identical visual inputs. The examples show that naive multimodal fusion does not consistently correct image-only errors and can introduce additional horizon-wise prediction errors. This supports the quantitative results in Table 5 and Table 7, where the multimodal model underperforms the image-only temporal model, particularly in high-risk recall.

## 5. Discussion

The experimental results provide several important insights.

First, under the current dataset and training setup, visual information emerges as the dominant modality for both semantic segmentation and danger-level prediction. As shown in Table 5 and Figure 3, the image-only temporal model consistently outperforms the weather-only and multimodal configurations across the main evaluation metrics.

Second, temporal modeling is essential for future risk prediction. As demonstrated in Table 7, removing temporal structure leads to substantial performance degradation, with Macro-F1 decreasing from 0.532 to 0.227 and high-risk recall decreasing to 0.000. This result highlights that temporal dependencies are critical for capturing evolving flood dynamics.

Third, multimodal learning is not always beneficial, particularly when the additional modality is noisy, weakly correlated with the target, or temporally misaligned with the primary visual evidence. As shown in Table 5 and Table 7, naive multimodal fusion does not improve performance and substantially reduces high-risk recall.

As illustrated in Figure 6, adding weather features through naive fusion does not consistently improve horizon-wise predictions over the image-only model under identical visual inputs.

One possible explanation is the imbalance between modalities. In the current dataset, visual features already provide strong and direct signals for flood risk, while meteorological features may have weaker or indirect correlations. Additionally, mismatches in feature scale or insufficient temporal alignment between modalities may further degrade fusion effectiveness. This interpretation is consistent with prior multimodal learning studies showing that feature-level fusion can be vulnerable to modality imbalance, heterogeneous noise, and weak cross-modal correspondence [8,9]. Therefore, future work should focus on cross-modal attention or adaptive fusion mechanisms to better align heterogeneous inputs.

This finding is important because multimodal sensor fusion is often assumed to improve prediction performance by providing complementary information [8,9]. However, our results show that this assumption does not always hold in real-world environmental sensing scenarios. When weather observations are collected from nearby AWS stations rather than from the exact CCTV sites, the environmental signals may not directly correspond to the visual flood conditions observed in the images. In addition, rainfall and river-level changes may influence CCTV-visible water regions with delayed or site-dependent responses, as commonly observed in hydrological forecasting contexts [1,2]. Under a small-data regime, these factors can cause the fusion model to learn unstable cross-modal correlations, resulting in poorer generalization than the image-only temporal baseline.

From a practical perspective, high-risk detection is one of the most critical objectives in disaster response because missed high-risk events can delay warning and mitigation actions [1,2]. In this regard, the image-only temporal model provides the most reliable performance under the current setting, making it a strong baseline for real-world deployment.

These findings are consistent with prior studies on multimodal learning, which report that naive fusion strategies can fail when modality imbalance, heterogeneous noise levels, or weak cross-modal correspondence exist [8,9]. Compared with conventional image-based flood-monitoring studies that mainly focus on flood detection, sensor-based monitoring, visual feature analysis, or current water-region segmentation [3,4,5,6], our study extends the task toward future danger-level forecasting by reformulating frame-level CCTV data into site-hour temporal sequences. In contrast to time-series-based hydrological prediction models that primarily rely on rainfall and water level measurements [1,2], the proposed framework explicitly evaluates the predictive role of CCTV-derived visual cues. The results show that, under noisy and weakly aligned environmental observations, visual temporal evidence can be more reliable than naive multimodal fusion for short-term high-risk detection.

Overall, the results highlight three key observations. First, temporal modeling is essential for future danger-level prediction, as Macro-F1 improves from 0.227 to 0.532 when temporal structure is incorporated. Second, naive multimodal fusion is not necessarily beneficial under noisy or weakly aligned environmental observations, as the multimodal model performs worse than the image-only temporal model. Third, the proposed relabeling and dataset reformulation strategy enables meaningful learning from the original dataset, which was otherwise limited by imbalanced labels, missing water level observations, and insufficient temporal organization. These findings support the practical value of the proposed framework for short-term CCTV-based flood-risk forecasting.

### Limitations

This study has several limitations. First, the reformulated dataset contains only 230 sequence samples. Although this reformulation improves temporal consistency and enables multi-horizon forecasting, the small number of sequences limits the generalization ability of deep models and increases the risk of overfitting, particularly for multimodal fusion. Second, the meteorological data are obtained from AWS observations that may not perfectly represent the local hydrological conditions visible in each CCTV scene. This spatial and temporal mismatch may partly explain why the naive multimodal model underperforms the image-only temporal model, consistent with prior findings that multimodal fusion can be sensitive to modality imbalance and weak cross-modal correspondence [8,9]. Third, the proposed relabeling strategy is empirical and domain-driven. While it improves label balance and interpretability, future work should validate the generated danger levels using physically measured water levels or expert annotations.

## 6. Conclusions

This study addressed CCTV-based flood-risk forecasting by reformulating static image-level flood monitoring into a temporal multi-horizon prediction task. The original dataset was not directly suitable for future risk prediction because it contained highly imbalanced danger-level annotations, largely missing water level observations, and insufficient temporal organization. To overcome these limitations, we introduced a site-relative relabeling strategy and transformed frame-level CCTV records into site-hour sequences. This reformulation enabled joint learning of current-frame semantic segmentation and future danger-level prediction at 1 h, 3 h, and 6 h horizons.

The experimental results demonstrate that the proposed reformulation provides a practical basis for predictive flood-risk analysis using CCTV-based environmental sensing. The temporal image-only model achieved the strongest overall forecasting performance under the current dataset setting, while maintaining meaningful segmentation capability for flood-related visual regions. In contrast, the naive multimodal model did not consistently improve performance over the image-only model. This result suggests that simply concatenating visual and meteorological features may be insufficient when environmental observations are noisy, weakly aligned with the CCTV scene, or only indirectly related to the visible flood state. Therefore, an important finding of this study is that multimodal fusion should be carefully designed and validated rather than assumed to improve forecasting performance.

From a practical perspective, the proposed framework demonstrates the potential of CCTV imagery as a useful source of environmental sensing information for short-term flood-risk forecasting and early-warning support. In particular, the results indicate that visually observable water regions and their temporal changes can provide meaningful cues for future danger-level prediction. The dataset relabeling and static-to-sequential transformation strategy also highlights the importance of task reformulation when existing public datasets are originally designed for current-state recognition rather than forecasting.

This study still has several limitations. First, the reformulated dataset contains a limited number of site-hour sequences, which restricts the generalization ability of deep models, especially under site-held-out or rare high-risk conditions. Second, weather observations may not perfectly match the local hydrological conditions observed in each CCTV image because of spatial and temporal misalignment between AWS stations and CCTV sites. Third, high-risk events remain relatively scarce, making reliable recognition of severe flood-risk states challenging. These limitations partly explain why naive multimodal fusion was sensitive to noise and did not consistently improve prediction performance.

Future work will focus on expanding the dataset across more CCTV sites, longer observation periods, and more diverse flood events. We will also investigate robust multimodal fusion strategies that explicitly account for cross-modal alignment, modality reliability, and site-specific characteristics. In addition, improving high-risk event recall, validating the relabeled danger levels with measured water level data or expert annotations, and evaluating real-time deployment scenarios will be important steps toward operational CCTV-based flood early-warning systems.

## Figures and Tables

**Table 1:** Statistics of the original dataset.

Site	#Number of Frames	Observational Water Level	River Level	Danger Level
Multiple sites	2861	Largely missing (>80%)	Highly imbalanced (majority class: 0)	Severely imbalanced (dominant class: 1, >70%)

**Table 2:** Comparison between the original and reformulated datasets.

Property	Original	Reformulated
Sample Unit	Frame	Site-hour
#Number of Samples	2861	230
Label Type	Static	Temporal
Danger Classes	Single	4 classes
Forecasting	No	Yes

**Table 3:** Class distribution before and after relabeling.

Danger Level	Original	Reformulated
0	2003	235
1	426	74
2	283	53
3	149	38

**Table 4:** Configuration of the proposed model.

Component	Description
Vision Encoder	SegFormer-B0
Weather Encoder	MLP + Temporal Aggregation
Fusion	Feature Concatenation + Convolution
Tasks	Segmentation + Forecasting
Horizons	1 h, 3 h, 6 h

**Table 5:** Overall performance comparison.

Model	mIoU ↑	Dice ↑	Macro-F1 ↑	Acc ↑	MAE ↓	High-Risk Recall ↑
Image-only	**0.892**	**0.940**	**0.532**	**0.637**	**0.498**	**0.642**
Weather-only	0.000	0.000	0.289	0.581	0.684	0.205
Multimodal	0.884	0.936	0.267	0.558	0.650	0.070

↑ indicates that higher values are better, whereas ↓ indicates that lower values are better. Bold values indicate the best performance among the compared models.

**Table 6:** Temporal consistency before and after dataset reformulation.

Dataset	Unit	Label Diversity	Temporal Consistency	Forecasting Support
Original	Frame	Low	Inconsistent	No
Reformulated	Site-hour sequence	Improved	Improved	Yes

**Table 7:** Ablation study on temporal modeling and modality configuration.

Model Variant	Macro-F1 ↑	High-Risk Recall ↑
No temporal/no risk modeling	0.227	0.000
Weather-only	0.289	0.205
Multimodal	0.267	0.070
Image-only temporal	**0.532**	**0.642**

↑ indicates that higher values are better. Bold values indicate the best performance among the compared model variants.

**Table 8:** Sensitivity analysis of relabeling weight variants.

Variant	V. wts	W. wts	Macro-F1 ↑	High-Risk Recall ↑	Note
Default	3.0/2.0	0.3/0.2	0.5066	0.6333	Visual-dominant baseline
Reduced weather	3.0/2.0	0.1/0.1	0.4628	0.4643	Weather sensitivity
Increased weather	3.0/2.0	0.6/0.4	0.4616	0.4333	Weather over-reliance
Visual only	3.0/2.0	0/0	0.3439	0.4706	Visual-only scoring

↑ indicates that higher values are better.

## Data Availability

The data used in this study are publicly available from AIHUB. The processed dataset and code used for this study are available from the corresponding author upon reasonable request.
